# Supplementary material for: Neutrophil-specific transcriptomic profiling reveals a novel signature for active tuberculosis diagnosis
Source: Microbiol Spectr. 2026 Mar 12;14(4):e01915-25. doi: 10.1128/spectrum.01915-25 (PMC13055310; doi:10.1128/spectrum.01915-25)
Supplement: Supplemental material — Fig. S1 to S6; Tables S1 and S2. [file spectrum.01915-25-s0001.docx]

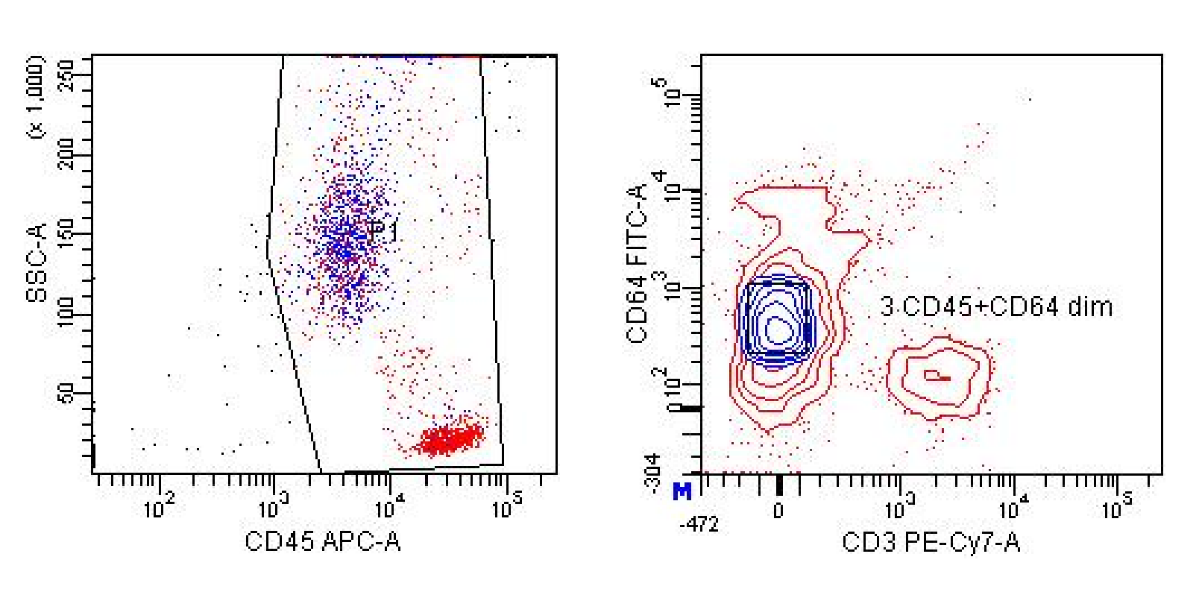


Figure S1. Gating strategy to access CD64+ neutrophils. Lymphocytes were gated using side scatter (SSC) parameter, and CD64+ subsets were identified as CD45-positive, CD3-negative and CD64 dim.


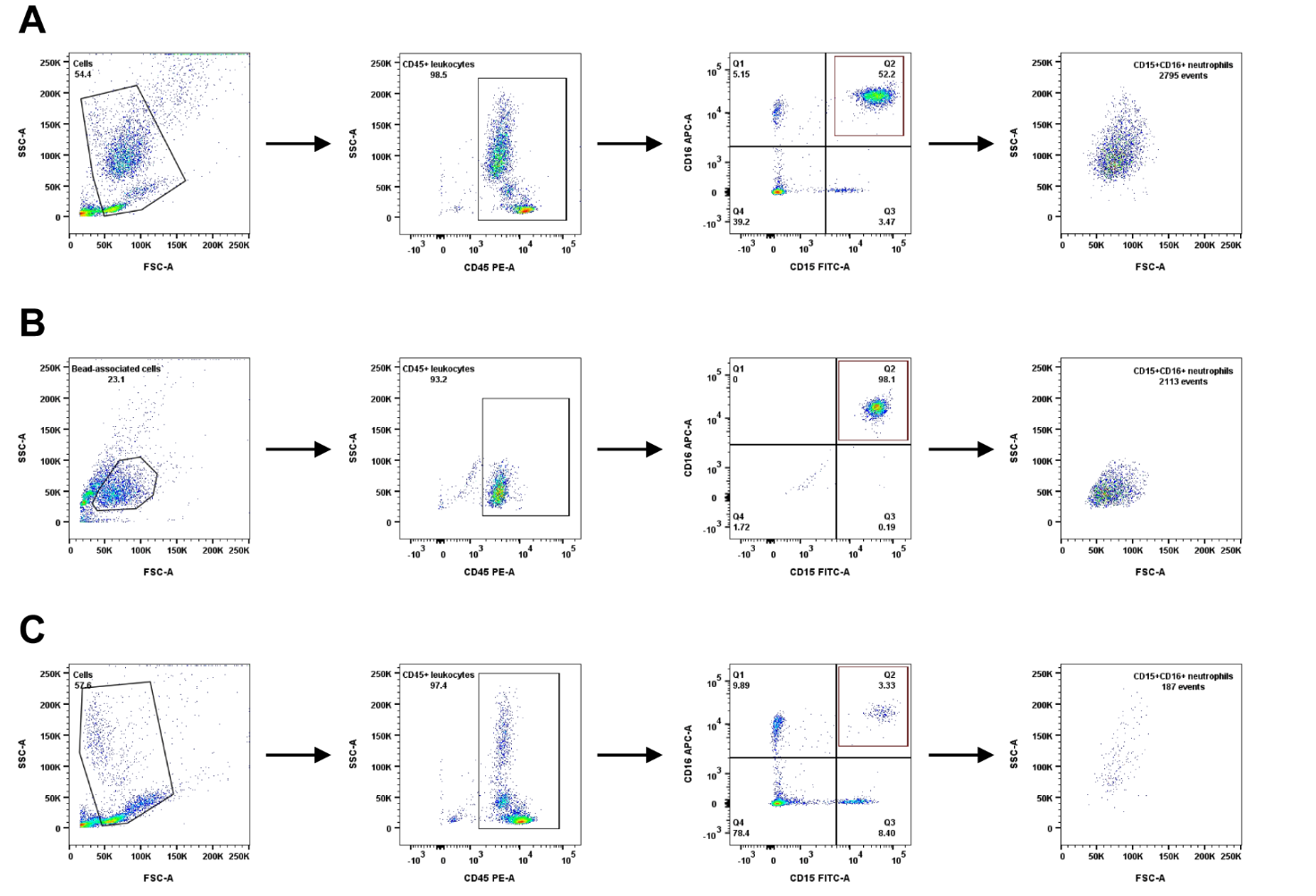


Figure S2. Flow-cytometric purity assessment of CD15-selected neutrophils. (A) Baseline: 52.2 % of CD45+ leukocytes in whole blood were CD15+CD16+ neutrophils. (B) Post-selection fraction: 98.1 % of CD45+ events in the enriched product corresponded to this subset. (C) Waste fraction: only 3.3 % of the recovered cells remained CD15⁺CD16⁺, indicating > 95 % target-cell recovery and >90% enriched purity. Data were acquired on a BD FACS Canto II (BD Biosciences) and analyzed with FlowJo v10. CD15 micro-bead enrichment was performed with the Thermo Fisher™ Neutrophil Isolation Kit according to the manufacturer’s instructions.


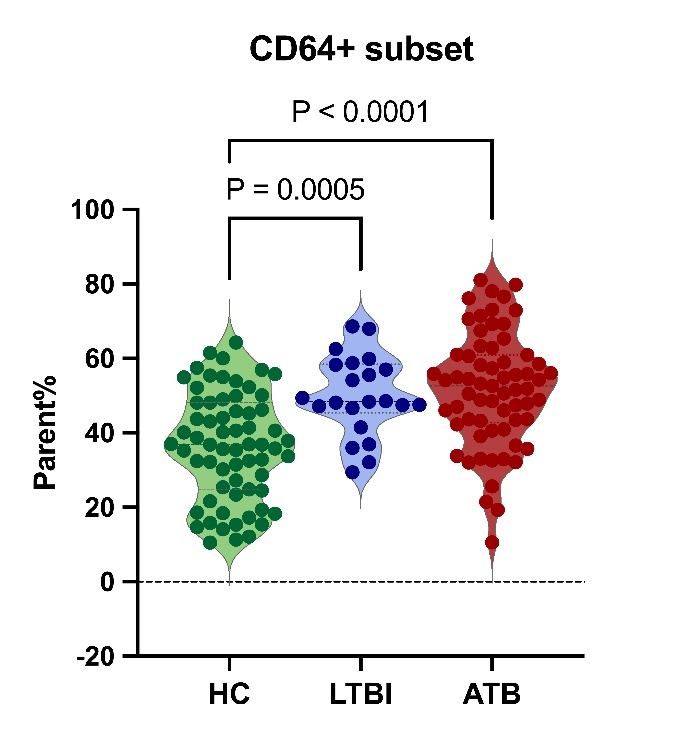


Figure S3. Statistical analysis of CD64⁺ subset expansion across participant groups in Discovery Cohort 1. The numbers of CD64⁺ cells were significantly higher in both ATB and LTBI groups than in HC (p < 0.0001), indicating that expansion of the CD64⁺ subset is closely associated with *M. tuberculosis* infection and disease status.


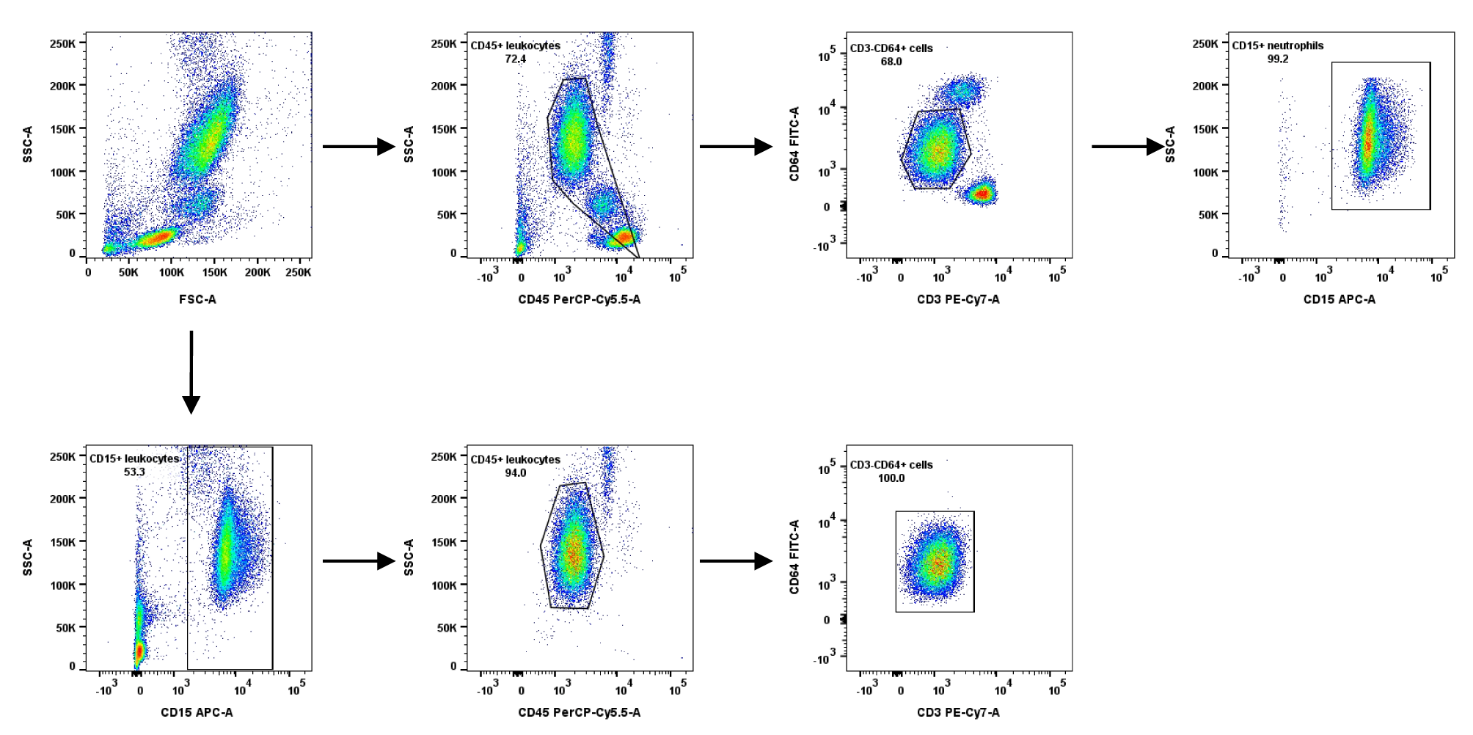


Figure S4. Flow-cytometric analysis of lymphocytes showing simultaneous surface expression of CD15 and CD64. Upper panel: After gating on CD45⁺CD64⁺CD3⁻ events (anti-CD45 PerCP-Cy5.5, anti-CD64 FITC and anti-CD3 PE-Cy7), 99.2 % of CD64-positive cells co-expressed CD15 (anti-CD15 APC). Bottom panel: 94.0 % of CD15-positive cells within the identical gate were also positive for CD64.


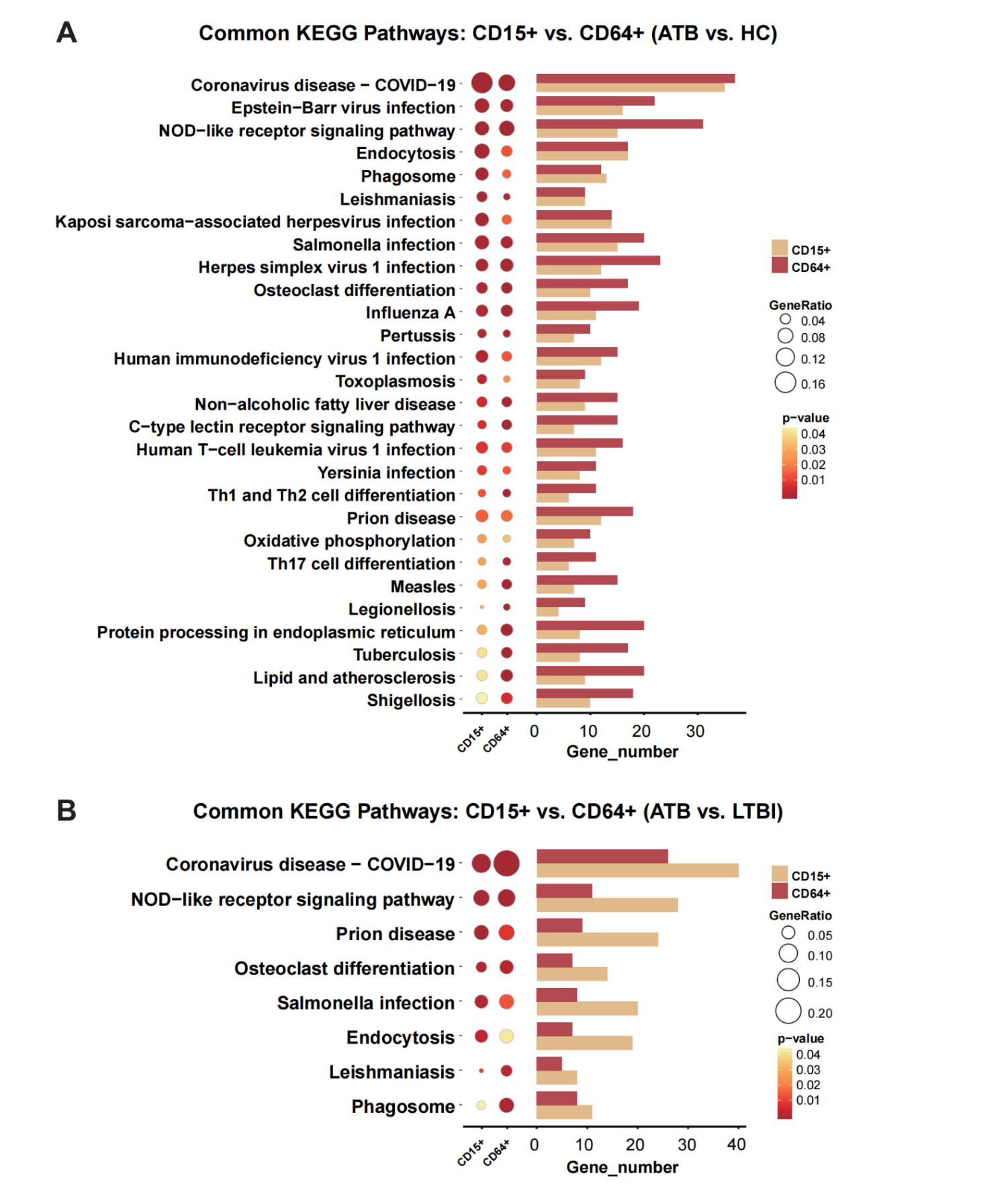


Figure S5. Common KEGG pathways in CD64⁺ and CD15⁺ subsets. A. ATB vs HC: 28 pathways shared, containing 258 DEGs (p < 0.05, |logFC| > 0.5); these represent 60 % of the DEGs detected in the CD64⁺ subset and 37 % of those in the CD15⁺ subset. B. ATB vs LTBI: 8 pathways shared, containing 170 DEGs; these represent 69 % of the DEGs in the CD64⁺ subset and 24 % of those in the CD15⁺ subset.


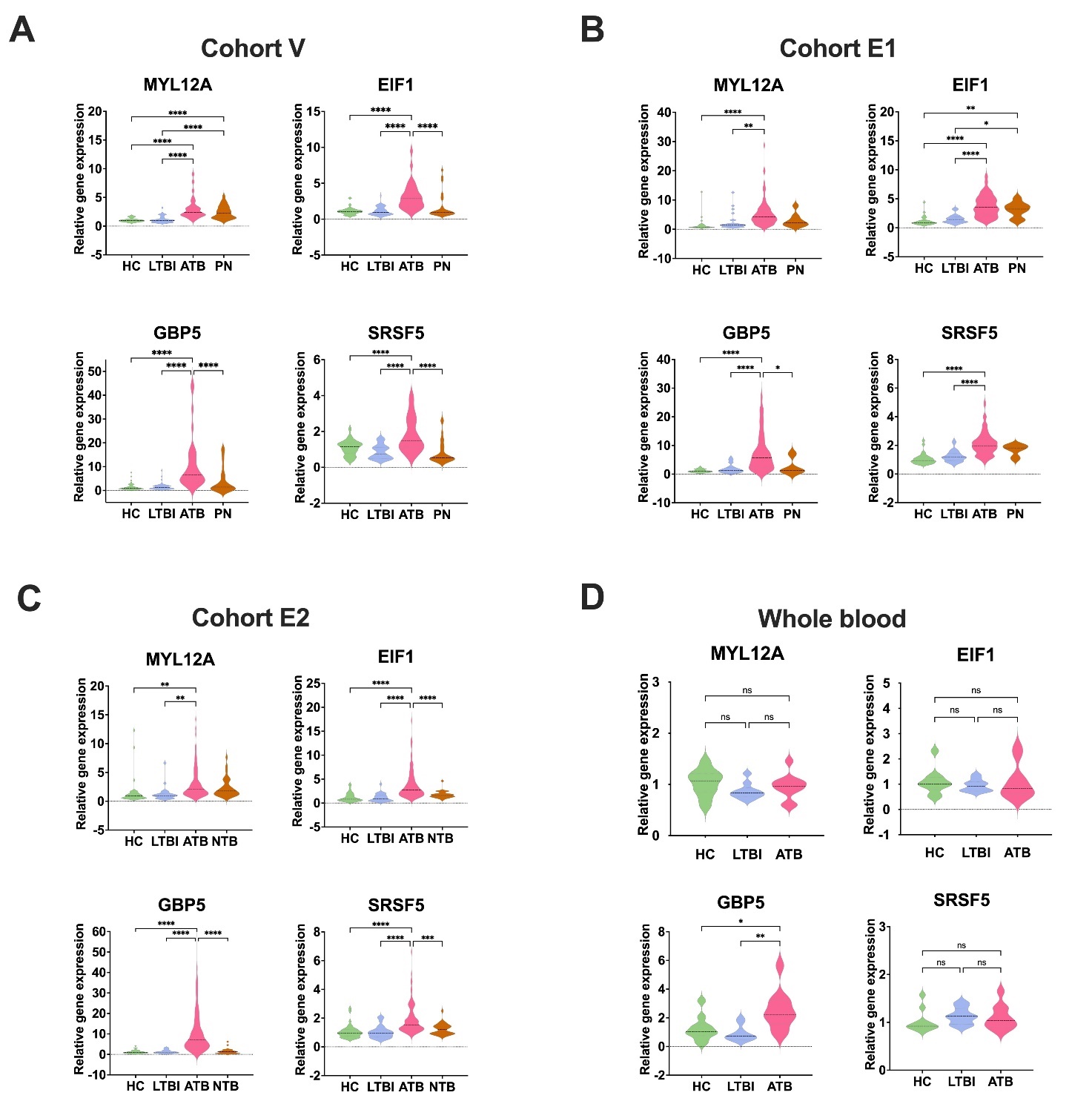


Figure S6. Statistical analysis of gene expression level detected by qPCR reaction. A, B, C, D refer to comparisons of gene expressions (MYL12A, EIF1, GBP5 and SRSF5) among ATB, LTBI and HC in different cohorts. “*”, “**”, “***”and “****” means p<0.05, 0.01, 0.005 and 0.001, respectively.

Table S1: TaqMan® probes for gene expression assays:

| Genes | Catalog NO. | Assay ID |
| --- | --- | --- |
| MYL12A | 4351372 | Hs07287167_m1 |
| EIF1 | 4351372 | Hs03044156_g1 |
| GBP5 | 4331182 | Hs00369472_m1 |
| SRSF5 | 4351372 | Hs00951033_g1 |
| NFE2 | 4351372 | Hs00973757_m1 |
| THEMIS2 | 4351372 | Hs00982814_g1 |
| MYO1F | 4448892 | Hs01027595_m1 |

Table S2. p-Value and log₂FC thresholds of the 20 candidate genes

| **Gene_id** | | **Protein** | **GO Function Categories^a^** | **CD15+neutrophil RNA-seq** | | | | | | | **CD64+neutrophil RNA-seq** | | | | | |
| --- | --- | --- | --- | --- | --- | --- | --- | --- | --- | --- | --- | --- | --- | --- | --- | --- |
|  |  |  |  | **ATB vs HC** | | | **ATB vs LTBI** | | | | **ATB vs HC** | | | **ATB vs LTBI** | | |
|  |  |  |  | **log_2_FC** | **p-value** | **padj** | **log_2_FC** | **p-value** | **padj** | **log_2_FC** | | **p-value** | **padj** | **log_2_FC** | **p-value** | **padj** |
| AKAP13 | A-kinase anchor protein 13 | | molecular adaptor activity | -0.194 | 0.113 | 0.509 | -0.205 | 0.076 | 0.372 | 0.995 | | 0.607 | 0.759 | 0.847 | 0.137 | 0.645 |
| BST1 | | ADP-ribosyl cyclase_cyclic ADP-ribose hydrolase 2 | immune response | 0.000 | 1.000 | 1.000 | -0.060 | 0.830 | 0.952 | 0.976 | | 0.000 | 0.004 | 0.977 | 0.003 | 0.280 |
| CARD16 | | Caspase recruitment domain-containing protein 16 | response to external biotic stimulus | 0.648 | 0.010 | 0.149 | 1.001 | 0.000 | 0.003 | 1.042 | | 0.000 | 0.009 | 0.807 | 0.008 | 0.357 |
| CCNL1 | | DNA replication licensing factor MCM2 | regulation of protein metabolic process | 1.161 | 0.000 | 0.000 | 1.106 | 0.000 | 0.000 | 1.087 | | 0.620 | 0.769 | 0.879 | 0.971 | 0.991 |
| CDA | | Cytidine deaminase | response to external biotic stimulus | -0.008 | 0.972 | 0.995 | 0.133 | 0.622 | 0.872 | 0.941 | | 0.001 | 0.036 | 0.760 | 0.011 | 0.370 |
| CHMP2A | | Charged multivesicular body protein 2a | biological process involved in interspecies interaction between organisms | 0.142 | 0.269 | 0.702 | 0.537 | 0.000 | 0.008 | 1.494 | | 0.037 | 0.236 | 1.037 | 0.573 | 0.863 |
| CLEC2B | | C-type lectin domain family 2 member B | defense/immunity protein | 0.868 | 0.000 | 0.001 | 1.155 | 0.000 | 0.000 | 0.821 | | 0.000 | 0.004 | 0.476 | 0.029 | 0.475 |
| EIF1 | | Eukaryotic translation initiation factor 1 | Translation | 1.002 | 0.000 | 0.000 | 0.926 | 0.000 | 0.000 | 1.141 | | 0.091 | 0.307 | 1.130 | 0.250 | 0.736 |
| GBP5 | | Guanylate-binding protein 5 | response to external biotic stimulus | 2.042 | 0.000 | 0.000 | 2.444 | 0.000 | 0.000 | 2.466 | | 0.000 | 0.000 | 1.818 | 0.000 | 0.119 |
| HIST1H2BC | | Histone H2B type 1A | protein-containing complex assembly | 0.955 | 0.000 | 0.005 | 1.069 | 0.000 | 0.003 | 1.169 | | 0.002 | 0.061 | 0.646 | 0.155 | 0.664 |
| ITM2B | | Integral membrane protein 2B | regulation of protein metabolic process | -0.095 | 0.516 | 0.851 | 0.152 | 0.228 | 0.585 | 0.662 | | 0.000 | 0.023 | 0.785 | 0.002 | 0.280 |
| MSRB1 | | Methionine-R-sulfoxide reductase B1 | response to external biotic stimulus | 0.235 | 0.286 | 0.718 | 0.234 | 0.238 | 0.596 | 0.842 | | 0.001 | 0.031 | 0.556 | 0.100 | 0.613 |
| MYL12A | | Myosin regulatory light chain 12A | response to external stimulus | -0.140 | 0.339 | 0.750 | 0.349 | 0.012 | 0.141 | 0.677 | | 0.000 | 0.018 | 0.758 | 0.000 | 0.132 |
| NFE2 | | Transcription factor NF-E2 45 kDa subunit | response to external stimulus | -0.250 | 0.337 | 0.749 | -0.405 | 0.086 | 0.392 | 0.775 | | 0.001 | 0.033 | 0.227 | 0.297 | 0.780 |
| ODF3B | | Outer dense fiber protein 3B | structure protein | 0.880 | 0.003 | 0.080 | 1.003 | 0.001 | 0.029 | 1.290 | | 0.000 | 0.006 | 0.288 | 0.450 | 0.841 |
| SECTM1 | | Secreted and transmembrane protein 1 | immune response | 0.925 | 0.000 | 0.001 | 0.919 | 0.000 | 0.001 | 0.891 | | 0.000 | 0.015 | 0.624 | 0.028 | 0.475 |
| SH3KBP1 | | SH3 domain-containing kinase-binding protein 1 | regulation of response to stimulus | -0.163 | 0.190 | 0.625 | 0.022 | 0.862 | 0.962 | 0.844 | | 0.982 | 0.991 | 0.981 | 0.886 | 0.968 |
| SRSF5 | | Serine_arginine-rich splicing factor 5 | response to organic substance | 0.873 | 0.000 | 0.000 | 1.106 | 0.000 | 0.000 | -0.260 | | 0.185 | 0.413 | -0.515 | 0.115 | 0.628 |
| STAT1 | | Signal transducer and activator of transcription 1-alpha_beta | response to external biotic stimulus | 0.750 | 0.000 | 0.004 | 1.013 | 0.000 | 0.000 | 1.026 | | 0.000 | 0.002 | 0.443 | 0.046 | 0.522 |
| THEMIS2 | | protein THEMIS2 | defense response | 0.180 | 0.318 | 0.739 | 0.370 | 0.027 | 0.223 | 0.696 | | 0.001 | 0.038 | 0.078 | 0.147 | 0.659 |

According to results of GO annotations (ref. 15)
